# Duodenal Ulcer with Massive Gastrointestinal Hemorrhage as an Initial Manifestation in Multiple Myeloma with Extramedullary Disease: A Case Report

**DOI:** 10.3390/medicina58010134

**Published:** 2022-01-16

**Authors:** Hao-Tse Chiu, Po-Huang Chen, Hao Yen, Chao-Yang Chen, Chih-Wei Yang, Yu-Hong Liu, Wu-Feng Hsieh, Shih-Hao Chou, Ta-Wei Pu

**Affiliations:** 1 Department of Surgery, Tri-Service General Hospital, National Defense Medical Center, Taipei 11490, Taiwan; haotsechiu@gmail.com (H.-T.C.); Cartilage77@yahoo.com.tw (C.-Y.C.); lovewithoutreasons@gmail.com (Y.-H.L.); howard0527@gmail.com (S.-H.C.); 2 Department of Internal Medicine, Tri-Service General Hospital, National Defense Medical Center, Taipei 11490, Taiwan; chenpohuang@hotmail.com; 3 Department of Pathology, Tri-Service General Hospital, National Defense Medical Center, Taipei 11490, Taiwan; kastyplanet@gmail.com; 4 Division of Colon and Rectal Surgery, Tri-Service General Hospital, National Defense Medical Center, Taipei 11490, Taiwan; 5 Department of Medicine, Division of Gastroenterology, Tri-Service General Hospital, National Defense Medical Center, Taipei 11490, Taiwan; youngwayleon@gmail.com; 6 Department of Anesthesiology, Tri-Service General Hospital, School of Medicine, National Defense Medical Center, Taipei 11490, Taiwan; hsiehwufeng@gmail.com; 7 Department of Surgery, Division of Colon and Rectal Surgery, Songshan Branch, Tri-Service General Hospital, National Defense Medical Center, Taipei 11490, Taiwan

**Keywords:** gastrointestinal hemorrhage, small bowel obstruction, multiple myeloma, extramedullary plasmacytoma

## Abstract

Plasma cell neoplasms are characterized by dysregulated proliferation of mature B cells, which can present with either single (solitary plasmacytoma) or systemic (multiple myeloma (MM)) involvement. MM with extramedullary plasmacytoma (EMP) is a rare disease that accounts for approximately 3–5% of all plasmacytomas. EMP with gastrointestinal (GI) system involvement is an even rarer entity, accounting for <1% of MM cases. We present a case of aggressive MM with EMP invading the duodenum, initially presented with massive upper GI hemorrhage and small bowel obstruction. A 67-year-old woman was admitted to our hospital owing to a lack of either gas or feces passage for 3 days. Abdominal distention and vomit with a high coffee ground content were observed for 24 h. The patient’s condition was initially diagnosed as small bowel obstruction, upper gastrointestinal bleeding, severe anemia, acute renal failure, and hypercalcemia. Furthermore, an analysis of immunoelectrophoresis in the blood, bone marrow aspiration, and tissue biopsy supported the diagnosis of MM and EMP invading the duodenum, upper GI hemorrhage, and small bowel obstruction. Our study provided the possible involvement of MM and EMP in the differential diagnosis of patients with unexplained GI hemorrhage and small bowel obstruction. A thorough review of the literature regarding the association between MM, GI hemorrhage, and small bowel obstruction is presented in this study.

## 1. Introduction

Upper gastrointestinal (GI) hemorrhage is a common medical condition with various etiologies, including peptic ulcer disease, esophagogastric varices, and primary GI tract neoplasms. However, some hematological diseases manifesting as GI hemorrhage may be ignored clinically. Hematological diseases account for approximately 10% of patients with GI hemorrhage. The pathogenesis of GI hemorrhage caused by hematological diseases is usually multi-factorial, such as mucosal damage due to malignant hematopoietic cell infiltration, bone marrow suppression, and infection caused by an immunocompromised status [1]. The typical characteristic of multiple myeloma (MM) is the neoplastic proliferation of plasma cells in the bone marrow, with the overproduction of monoclonal immunoglobulin. Anemia, bone pain, renal dysfunction, fatigue, and hypercalcemia are the most common symptoms of this disease. Although most patients with MM only have intramedullary involvement, extramedullary plasmacytomas (EMPs) are observed in approximately 6–8% of patients with MM at the time of diagnosis, which are often located in the head and neck region [2]. EMPs with GI tract involvement are still uncommon, accounting for <1% of MM cases. Herein, we present a case of aggressive MM with EMP invading the duodenum, and presenting with upper GI hemorrhage and small bowel obstruction.

## 2. Case Report

**Figure 1:**
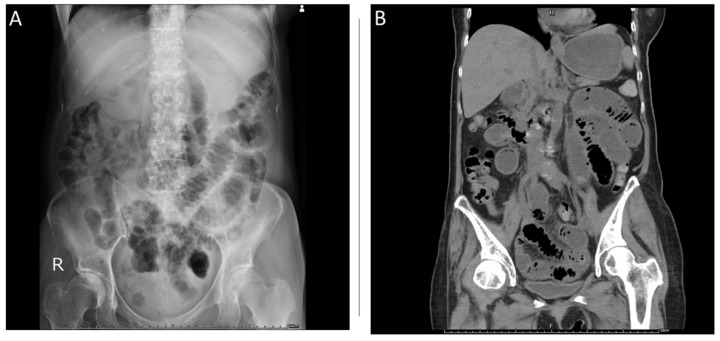
Abdominal plain view computed tomography (CT). (**A**) Abdominal plain view CT showing distention of small bowel loops with a coiled spring sign. (**B**) Abdominal CT showing generally dilated, air-fluid levels in the intestine, compatible with small intestinal obstruction.

**Figure 2:**
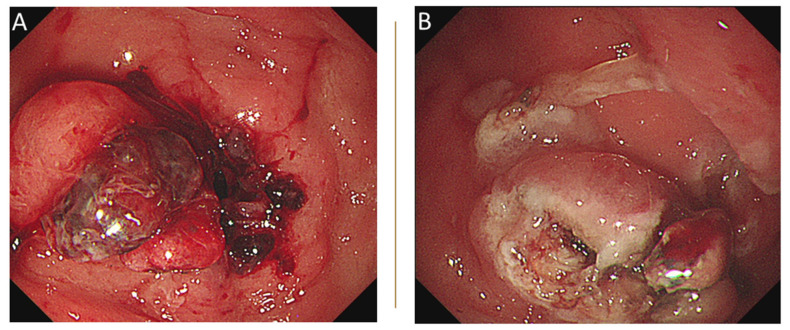
Endoscopy of the duodenum. (**A**) Endoscopy showing a polypoid lesion with a central A1 ulcer in the duodenum with active oozing. (**B**) Treatment with endoscopic electrocoagulation of the lesion.

**Figure 3:**
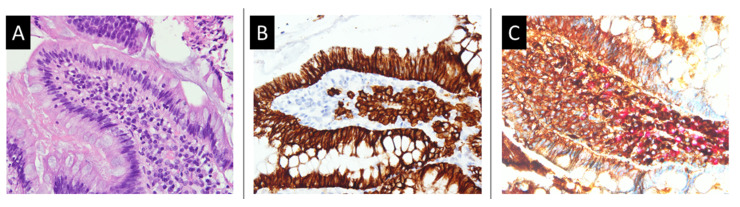
Histology findings of the endoscopic biopsied tissues. (**A**) Plasma cells aggregate in the lamina propria of the duodenal mucosa (hematoxylin–eosin staining, 200×). (**B**) CD138 immunohistochemical staining highlighting plasma cells in the lamina propria. The highlighted plasma cells are brown inside the lamina propria (immunohistochemical staining, 200×). (**C**) The kappa/lambda light chain double staining shows two colors (dark brown: kappa chain; red: lambda chain), revealing polyclonality of the kappa and lambda light chains (immunohistochemical staining, 200×).

**Figure 4:**
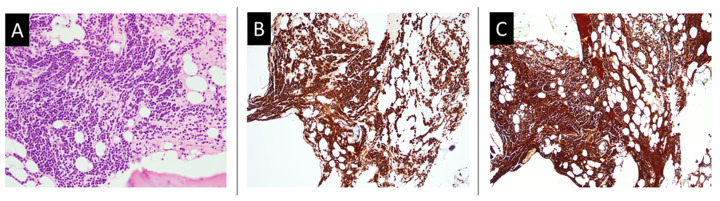
Histology findings of bone marrow biopsy. (**A**) Abundant plasma cells aggregate in the bone marrow tissue, replacing the normal distribution of bone marrow cells (hematoxylin–eosin staining, 100×). (**B**) CD138 immunohistochemical staining showing dark brown aggregating plasma cells (immunohistochemical staining, 40×). (**C**) The kappa/lambda light chain double staining shows a single color (brown) of the stained cells, indicating kappa light chain monoclonality. The normal polyclonality of the kappa and lambda light chains may show two colors (brown and red, usually presenting kappa and lambda chains, respectively). The normal ratio of kappa cells and lambda cells is usually 2:1 (immunohistochemical staining, 40×).

A 67-year-old woman visited our hospital due to a lack of either gas or feces passage for 3 days. Abdominal distention and vomit with a high coffee ground content were observed for 24 h. She did not report any history of bleeding, abdominal operation, or use of anti-thrombotic agents or non-steroidal anti-inflammatory drugs. Physical examination revealed mild tenderness around the epigastric region, with slightly increased muscle tone without rebound tenderness. Abdominal auscultation revealed normal bowel sounds (approximately 8 per min). An abdominal plain view showed distension of the small bowel loops, with a coiled spring sign. Abdominal computed tomography revealed a small intestinal obstruction (Figure 1). Laboratory tests conducted at the emergency department revealed the following: white blood cell count of 6.6 k/mm^3^ (4.2–10.3), hemoglobin content of 6.2 g/dL (normal range: 12–16), platelet count of 318 k/mm^3^ (150–410), BUN of 31.4 mmol/L (2.5–6.1 mmol/L), SCr of 406.6 mmol/L (46–92 mmol/L, baseline), blood calcium content of 3.45 mmol/L (2.10–2.55), prothrombin time of 15.4 s (11.0–15.0 s), internationalized normalized ratio of 1.1, and a strong positive gastric juice occult blood level (negative). The patient’s condition was initially diagnosed as small bowel obstruction, upper gastrointestinal bleeding, severe anemia, acute renal failure, and hypercalcemia. The initial treatment consisted of nil per os with bowel rest, gastrointestinal decompression, intravenous fluid supplementation, blood transfusion, proton-pump inhibitor therapy, and salmon calcitonin therapy. In addition, the patient underwent emergency hemodialysis. Gastroscopy was performed on the fourth day of hospitalization, after bowel passage was restored; it showed a polypoid lesion with a central A1 ulcer in the duodenum with active oozing. Endoscopic electrocoagulation was performed on the oozing lesion with successful hemostasis (Figure 2). A biopsy of the duodenal ulcer and tumor showed plasma cell infiltration (Figure 3). Additionally, an immunofixation study demonstrated IgG plus kappa chain dominance on immunoelectrophoresis (IEF), and showed beta-2 microglobulin levels over 10 mg/dL (2.5–6.1 mmol/L). Based on these laboratory examinations, a bone marrow needle biopsy was performed, which revealed an increase in plasma cells (35%), and kappa monoclonality was identified in the bone marrow aspirate (Figure 4). Therefore, this patient was finally diagnosed with MM, IgG type, ISS criteria stage III, and Durie–Salmon criteria stage III. Further therapy was provided by the Department of Hematology and Oncology in our hospital after the symptoms of GI hemorrhage and small obstruction abated. However, the patient did not receive complete treatment for MM with EMP due to the occurrence of aspiration pneumonia with septic shock, leading to a sudden cardiac arrest on the 10th day of hospitalization. Therefore, there were no available follow-up data to evaluate the treatment effects.

## 3. Discussion

Plasma cell neoplasms are characterized by the dysregulated proliferation of mature B cells, which can present with either single (solitary plasmacytoma) or systemic (MM) involvement. MM with extramedullary plasmacytoma (EMP) is a rare disease that accounts for approximately 3–5% of all plasmacytomas [3]. EMP in the GI tract is rare, and the most common site is the small bowel, followed by the stomach, colon, and esophagus, according to limited case reports. The infrequent manifestation of GI hemorrhage and small bowel obstruction in EMP might easily lead the clinician to make an erroneous diagnosis. Several possible explanations have been reported regarding GI hemorrhage in patients with MM or EMP. First, the deposition of amyloid proteins in the GI wall leads to increased capillary fragility. Additionally, myeloma cells may directly infiltrate the GI tract in the form of plasmacytoma, and subsequently cause mucosal hyperplasia, edema, erosion, and repair dysfunction [4,5]. Furthermore, defective primary hemostasis was observed in patients with high serum β2-microglobulin and serum-free light chains, suggestive of a very active disease, which may be reflective of increased bleeding tendencies, as explained by Hinterleitner et al. [6]. Small bowel obstruction may also be involved in patients presenting with EMP, causing tethering of the bowel loops owing to plasma cell infiltration into the peritoneum [7,8]. Meanwhile, previous studies revealed that hypercalcemic crisis resulted in paralytic ileus, which may be a deteriorating factor contributing to decreased bowel motion [9,10]. Overall, a duodenal ulcer combined with GI hemorrhage and small bowel obstruction is rare, and has not been reported in previous studies.

MM with EMP remains a treatment challenge without a treatment consensus and re-quires individual therapy [11]. Moreover, studies about the treatment of MM with EMP-related GI bleeding are rare. In terms of local treatment, complete surgical resection for symptomatic EMP is regarded to control the symptoms, while radiotherapy may be performed to treat unresectable lesions [2,12]. On the other hand, systemic treatment and induction therapy should be initiated promptly in a patient with MM and EMP [13]. The initial therapy for patients with symptomatic MM depends on risk stratification, eligibility for autologous hematopoietic cell transplantation, and available resources. In this case, endoscopic electrocoagulation was performed as an emergent local treatment of GI hemorrhage with successful hemostasis. In general, MM is a widespread disease and is sometimes accompanied by EMP. The choice of treatment is very complicated and should be based on different patient factors. Non-hematologists may not have a sufficient understanding about MM and may misdiagnose patients.

## 4. Conclusions

Knowledge of differential diagnoses of MM with EMP is crucial for treating patients with GI hemorrhage combined with small bowel obstruction, especially those with other traditional abnormalities on laboratory tests, such as severe anemia, acute kidney injury, and hypercalcemia.

## Data Availability

All data regarding the findings are available within the manuscript.
